# New frontiers in radiologic interpretation: evaluating the effectiveness of large language models in pneumothorax diagnosis

**DOI:** 10.1371/journal.pone.0331962

**Published:** 2025-09-12

**Authors:** Bensu Bulut, Medine Akkan Öz, Murat Genç, Ayşenur Gür, Mehmet Yortanlı, Betül Çiğdem Yortanlı, Oguz Sariyildiz, Ramiz Yazıcı, Hüseyin Mutlu, Mustafa Sirri Kotanoglu, Eray Cinar, Zekeriya Uykan

**Affiliations:** 1 Department of Emergency Medicine, Ankara Gulhane Training and Research Hospital, Health Science University, Ankara, Turkey; 2 Department of Emergency Medicine, Ankara Training and Research Hospital, Ankara, Turkey; 3 Department of Emergency Medicine, Etimesgut Şehit Sait Ertürk State Hospital, Ankara, Turkey; 4 Department Of Emergency Medicine, Konya Numune Hospital, Konya, Turkey; 5 Department of Internal Medicine, Konya City Hospital, University of Health Sciences, Konya, Turkey; 6 Department of Anesthesıology And Reanımatıon Department, 75th Year Oral And Dental Health Hospital, Ankara, Turkey; 7 Department of Emergency Medicine, Istanbul Kanuni Sultan Suleyman Training and Research Hospital, Health Science University, Istanbul, Turkey; 8 Department of Emergency Medicine, Aksaray Training and Research Hospital, Aksaray University, Aksaray, Turkey; 9 Department of Anesthesıology And Reanımatıon Department, Ankara Training and Research Hospital, Ankara, Turkey; 10 Department of Thoracic Surgery, Bilkent City Hospital, University of Health Sciences, Ankara, Turkey; 11 College of Engineering and Technology, American University of the Middle East, Kuwait; University of Health Sciences, Beyhekim Training and Research Hospital, TÜRKIYE

## Abstract

**Background:**

This study evaluates the diagnostic performance of three multimodal large language models (LLMs)—ChatGPT-4o, Gemini 2.0, and Claude 3.5—in identifying pneumothorax from chest radiographs.

**Methods:**

In this retrospective analysis, 172 pneumothorax cases (148 patients aged >12 years, 24 patients aged ≤12 years) with both chest radiographs and confirmatory thoracic CT were included from a tertiary emergency department. Patients were categorized by age and pneumothorax size (small/large). Each radiograph was presented to all three LLMs accompanied by basic symptoms (dyspnea or chest pain), with each model analyzing each image three times. Diagnostic accuracy was evaluated using overall accuracy (all three responses correct), strict accuracy (≥2 responses correct), and ideal accuracy (≥1 response correct), alongside response consistency assessment using Fleiss’ Kappa.

**Results:**

In patients older than 12 years, ChatGPT-4o demonstrated the highest overall accuracy (69.6%), followed by Claude 3.5 (64.9%) and Gemini 2.0 (57.4%). Performance was significantly poorer in pediatric patients across all models (20.8%, 12.5%, and 20.8%, respectively). For large pneumothorax in adults, ChatGPT-4o showed significantly higher accuracy compared to small pneumothorax (81.6% vs. 42.2%; p < 0.001). Regarding consistency, Gemini 2.0 demonstrated excellent reliability for large pneumothorax (Kappa = 1.00), while Claude 3.5 showed moderate consistency across both pneumothorax sizes.

**Conclusion:**

This study, the first to evaluate these three current multimodal LLMs in pneumothorax identification across different age groups, demonstrates promising results for potential clinical applications, particularly for adult patients with large pneumothorax. However, performance limitations in pediatric cases and with small pneumothoraces highlight the need for further validation before clinical implementation.

## 1. Introduction

Pneumothorax is a life-threatening condition characterized by the partial or complete collapse of the lung due to the accumulation of air in the pleural cavity [1]. It is classified as spontaneous, traumatic, or iatrogenic, with reported incidence rates of 18–28 per 100,000 in men and 1.2–6 per 100,000 in women [2]. Primary spontaneous pneumothorax (PSP) can occur in healthy individuals without any underlying pulmonary pathology—referred to as primary spontaneous pneumothorax—or in association with chronic lung diseases such as Chronic Obstructive Pulmonary Disease (COPD), tuberculosis, or cystic fibrosis, in which case it is termed secondary spontaneous pneumothorax [3]. Clinically, it often presents with a sudden onset of chest pain and dyspnea, though symptom severity can vary depending on the size and rate of pneumothorax development [4]. Timely and accurate diagnosis in emergency departments is crucial in reducing both morbidity and mortality in acute conditions [5,6]. Radiological imaging plays a central role in the diagnosis of pneumothorax. Due to its accessibility and low radiation exposure, conventional chest radiography is typically the first-line imaging modality [7]. However, in cases involving small or complex pneumothoraces, thoracic computed tomography (CT) is considered the gold standard owing to its superior sensitivity and specificity. Still, it is not suitable as an initial diagnostic tool in every case due to its higher cost and radiation burden [8,9].

In recent years, the application of artificial intelligence (AI) and machine learning algorithms in medical imaging has gained considerable momentum. Deep learning-based image analysis systems have shown promising results in detecting pathological findings within radiological scans [10]. As highlighted by Zhang and Metaxas, fundamental AI models in image analysis offer the potential to enhance diagnostic accuracy while alleviating the workload of radiologists [9]. However, most existing systems focus solely on imaging data, lacking integration with clinical information. Emerging multimodal models such as ChatGPT, Claude, and Gemini are notable for their combined capabilities in both text-based reasoning and image interpretation [11,12]. Clusmann et al. emphasized in their study that large language models (LLMs) hold significant promise in healthcare, particularly for use in radiological assessment and clinical decision support systems, [13]. In this context, multimodal LLMs—capable of processing both image data and patient clinical information—offer a more holistic and integrated diagnostic approach. The use of AI to assess radiographic images is particularly impactful in emergency settings where rapid diagnosis is vital, and the availability of expert radiologists may be limited, [14].

Evaluating the diagnostic accuracy of multimodal LLMs in pneumothorax detection is crucial to understanding their potential role in clinical settings. Swift and accurate interpretation of radiographic images in emergency departments directly influences patient outcomes. Moreover, determining how these models perform across age groups and pneumothorax sizes can help identify scenarios in which they are most reliable.

This study aims to assess the diagnostic performance of three state-of-the-art multimodal large language models—ChatGPT-4o, Gemini 2.0, and Claude 3.5—in identifying pneumothorax, and to investigate how factors such as patient age and pneumothorax size affect diagnostic accuracy. The results will offer valuable insights into the clinical applicability of these models and help delineate areas for future development.

## 2. Materials and methods

### 2.1. Study design and participants

This retrospective study was conducted between March 6 and April 6, 2025, in the Emergency Medicine Department of Etlik City Hospital. Prior to initiation, the study received ethical approval from the Bilkent City Hospital Ethics Committee (Approval No: E2-25–10251), with a waiver for informed consent. The hospital operates as a tertiary care emergency center, managing approximately 60,000 patient admissions per month.

Patients who presented to the emergency department with complaints of dyspnea or chest pain and were diagnosed with pneumothorax based on both posteroanterior (PA) chest radiography and thoracic CT were included. Exclusion criteria involved patients with prior chest tube placement for pneumothorax, history of thoracic surgery, or cases where pneumothorax was not definitively confirmed by CT.

The authors accessed only anonymized lung imaging data from the hospital’s electronic records. No personally identifying information was available during or after data collection. Therefore, the requirement for informed consent was waived by the ethics committee.

Lung images were extracted from the hospital’s electronic medical records. Chest X-rays of patients with CT-confirmed PSP were independently reviewed and categorized as either “small” or “large” PSP by two authors: H.M. (20 years’ experience) and R.Y. (over 10 years’ experience). In cases of disagreement, classification was finalized by consensus with two additional experts, E.C. and M.S.K., both with more than 21 years of clinical experience.

For patients aged 12 years and older, the size classification followed the American College of Chest Physicians Delphi Consensus Statement: a pleural line-to-chest wall distance ≥3 cm at the apex was defined as a “large pneumothorax”; smaller distances were considered “small pneumothorax” [15]. In patients younger than 12 years, a pneumothorax affecting more than 15% of lung volume, as calculated by the Light Index, was classified as “large” [8,16–18]. Accordingly, all patients were grouped by pneumothorax size.

To ensure effective interpretation of the findings by LLMs, these models were primed using core textbooks in Emergency Medicine—Tintinalli’s Emergency Medicine: A Comprehensive Study Guide (9th Edition) and Rosen’s Emergency Medicine: Concepts and Clinical Practice (10th Edition)—as well as thoracic surgery references including General Thoracic Surgery (Shields) (9th Edition) and Holcomb and Ashcraft’s Pediatric Surgery (8th Edition). Between December 25 and 31, all image evaluations were performed on the same computer by M.A. Each case scenario, including the clinical context of a patient with dyspnea or chest pain, was presented once to ChatGPT-4o (OpenAI), Gemini 2.0 (Google DeepMind), and Claude 3.5 (Anthropic) models across three different days. Each model generated three independent responses per case, consistent with previous approaches aimed at assessing LLMs consistency and stability [19,20]. Diagnostic accuracy was assessed using three distinct criteria: overall Accuracy: all three responses were correct, strict Accuracy: at least two of the three responses were correct, ideal Accuracy: at least one of the three responses was correct.

### 2.2. Statistical analysis

All data analyses were conducted using IBM SPSS Statistics software (version 27.0, IBM Corp., Armonk, NY, USA). The distribution of continuous variables was evaluated using descriptive statistics, and categorical data were reported as frequencies and percentages (%). The diagnostic performance of the AI models in identifying pneumothorax was assessed based on three accuracy categories: strict accuracy (all three responses correct), overall accuracy (at least two responses correct), and ideal accuracy (at least one response correct). To determine whether there were statistically significant differences in diagnostic accuracy among the models for the same patient group, Cochran’s Q test was applied. In cases where Cochran’s Q test indicated significant differences, pairwise comparisons between models were performed using post hoc McNemar tests. Differences in diagnostic accuracy across age groups (under vs. over 12 years of age) and pneumothorax size (small vs. large) were evaluated using the Chi-square test; Fisher’s exact test was used when any cell frequency was less than 5. To assess the internal consistency of the responses provided by the same model across three trials per patient, Fleiss’ Kappa coefficient was calculated. A p-value <0.05 was considered statistically significant in all analyses.

## 3. Results

A total of 172 patients diagnosed with pneumothorax were included in the study, comprising 148 patients over the age of 12 and 24 patients under 12. In patients older than 12 years, the ChatGPT-4o model demonstrated a significantly higher strict accuracy in identifying large pneumothorax compared to small pneumothorax (81.6% vs. 42.2%; p < 0.001). For Gemini 2.0 and Claude 3.5, the difference in accuracy based on pneumothorax size was not statistically significant (p > 0.05). However, the Gemini 2.0 model showed significantly higher ideal accuracy in the large pneumothorax group (p = 0.019). Among patients younger than 12 years, none of the models exhibited statistically significant differences in diagnostic accuracy between small and large pneumothorax (p > 0.05) (Table 1).

**Table 1:** Comparison of Large Language Models’ Diagnostic Accuracy by Age Group and Pneumothorax Size.

	Age > 12			Age ≤ 12		
	**Small Pneumothorax** **(n = 45)**	**Large Pneumothorax (n = 103)**	**p**	**Small Pneumothorax** **(n = 10)**	**Large Pneumothorax (n = 14)**	**p**
Overall accuracy						
ChatGPT 4o	19 (42.2)	84 (81.6)	<0.001	2 (20.0)	3 (21.4)	0.932
Gemini 2.0	23 (51.1)	62 (60.2)	0.304	1 (10.0)	4 (28.6)	0.269
Claude 3.5	32 (71.1)	64 (62.1)	0.293	1 (10.0)	2 (14.3)	0.754
Strict accuracy						
ChatGPT 4o	37 (82.2)	93 (90.3)	0.167	4 (40.0)	6 (42.9)	0.889
Gemini 2.0	34 (75.6)	62 (60.2)	0.072	4 (40.0)	8 (57.1)	0.408
Claude 3.5	37 (82.2)	79 (76.7)	0.453	7 (70.0)	11 (78.6)	0.633
Ideal accuracy						
ChatGPT 4o	44 (97.8)	101 (98.1)	0.911	8 (80.0)	12 (85.7)	0.711
Gemini 2.0	36 (80.0)	62 (60.2)	0.019	7 (70.0)	11 (78.6)	0.633
Claude 3.5	42 (93.3)	90 (87.4)	0.283	8 (80.0)	12 (85.7)	0.711

**Fig 1:**
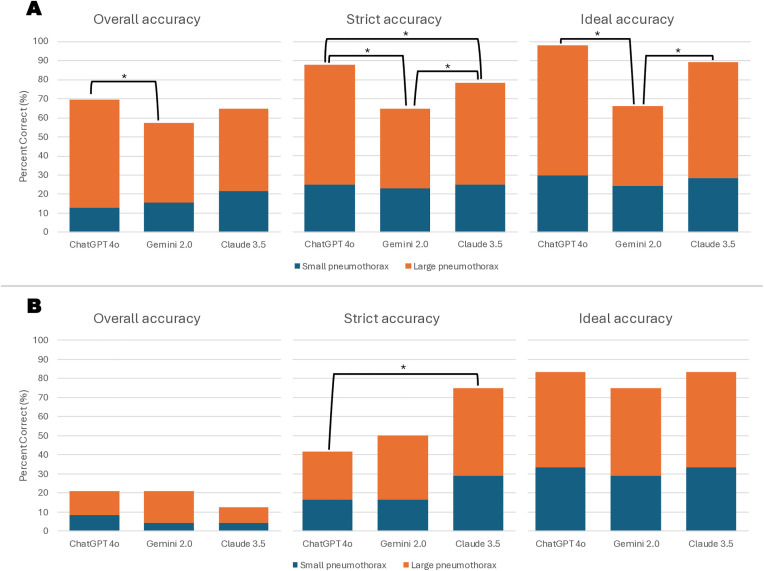
Comparison of diagnostic accuracy levels of different artificial intelligence models for pneumothorax detection across age groups. Panels A and B illustrate strict accuracy, overall accuracy, and ideal accuracy scores of the ChatGPT-4o, Gemini 2.0, and Claude 3.5 models in patients aged ≥12 and <12 years, respectively. Marked values indicate statistically significant differences between models (p < 0.05).

A statistically significant difference was observed among the models in patients aged 12 years and older. Regarding strict accuracy, the ChatGPT-4o, Gemini 2.0, and Claude 3.5 models achieved accuracies of 69.6%, 57.4%, and 64.9%, respectively, with this difference being statistically significant (p = 0.039). When comparing overall accuracy rates, the disparity was more pronounced (p < 0.001), and similarly, ideal accuracy rates also differed significantly between models (p < 0.001) (Table 2, Fig 1).

**Table 2:** Comparison of Diagnostic Accuracy of Artificial Intelligence Models Across Different Age Groups.

	ChatGPT 4o	Gemini 2.0	Claude 3.5	p
**Age > 12**;				
Overall accuracy	103 (69.6)	85 (57.4)	96 (64.9)	0.039
Strict accuracy	130 (87.8)	96 (64.9)	116 (78.4)	<0.001
Ideal accuracy	145 (98.0)	98 (66.2)	132 (89.2)	<0.001
**Age ≤ 12**;				
Overall accuracy	5 (20.8)	5 (20.8)	3 (12.5)	0.717
Strict accuracy	10 (41.7)	12 (50.0)	18 (75.0)	0.039
Ideal accuracy	20 (83.3)	18 (75.0)	20 (83.3)	0.717

Post hoc McNemar analyses following Cochran’s Q test revealed that, among patients aged 12 years and older, the ChatGPT-4o model demonstrated significantly higher strict accuracy compared to Gemini 2.0 (p = 0.021). In terms of overall accuracy, ChatGPT-4o outperformed both Gemini 2.0 (p < 0.001) and Claude 3.5 (p = 0.014). A statistically significant difference was also found between Gemini 2.0 and Claude 3.5 (p = 0.005). Regarding ideal accuracy, significant differences were observed between ChatGPT-4o and Gemini 2.0 (p < 0.001), as well as between Gemini 2.0 and Claude 3.5 (p < 0.001). In the under-12 age group, a significant difference in overall accuracy was identified between ChatGPT-4o and Claude 3.5 (p = 0.008).

The consistency of model responses was assessed using Fleiss’ Kappa coefficient, based on answers generated for the same patient’s image presented on three different occasions. Among patients aged 12 years and older, the ChatGPT-4o model exhibited moderate agreement in the large pneumothorax group (Kappa = 0.39; 95% CI: 0.28–0.50; p < 0.001), while consistency was low in the small pneumothorax group (Kappa = 0.04; p = 0.678). The Gemini 2.0 model demonstrated perfect agreement in the large pneumothorax group (Kappa = 1.00; 95% CI: 0.89–1.11; p < 0.001), and moderate consistency in the small pneumothorax group (Kappa = 0.55; p < 0.001). For the Claude 3.5 model, similar moderate levels of agreement were observed in both large and small pneumothorax groups (Kappa = 0.55 and 0.49, respectively; p < 0.001).

**Fig 2:**
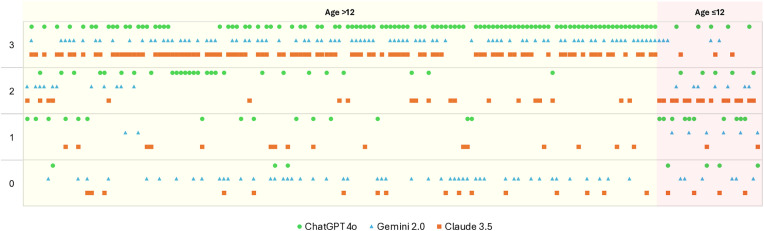
Distribution of AI Model Responses Across Diagnostic Accuracy Categories. This figure illustrates the distribution of response accuracy levels for ChatGPT-4o, Gemini 2.0, and Claude 3.5, based on three responses generated per question. Each model’s outputs were classified into four accuracy categories: Strict Accuracy (3 points) – all three responses correct; Overall Accuracy (2 points) – at least two correct; Ideal Accuracy (1 point) – at least one correct; and Incorrect (0 points) – all three responses incorrect.

In the subgroup of patients under 12 years old, consistency across model responses was generally low. For ChatGPT-4o, the Kappa coefficients were calculated as 0.20 (p = 0.282) for small pneumothorax and 0.14 (p = 0.355) for large pneumothorax. The Gemini 2.0 model showed statistically significant agreement only in the large pneumothorax group (Kappa = 0.33; p = 0.034). In contrast, the Claude 3.5 model yielded very low consistency in both groups, with no statistically significant differences observed (Fig 2).

In patients older than 12 years, all three models predominantly produced strictly accurate outputs, with ChatGPT-4o emerging as the most reliable among the models. In contrast, the distribution shifted toward lower accuracy categories in pediatric patients, with an increased proportion of partially correct or incorrect outputs. This pattern suggests that while the models provided more reliable results in adult patients, their performance was less consistent in pediatric cases (Fig 2).

## 4. Discussion

The capability of LLMs to interpret medical imaging is emerging as a transformative area with the potential to revolutionize clinical diagnosis and treatment pathways. In this study, we evaluated the diagnostic performance of three distinct LLMs—ChatGPT-4o, Gemini 2.0, and Claude 3.5—in detecting pneumothorax in patients presenting to the emergency department with dyspnea or chest pain. To our knowledge, this is the first study to assess pneumothorax imaging interpretations by these models across different age groups. Our findings demonstrated that in patients over 12 years of age, ChatGPT-4o, Gemini 2.0, and Claude 3.5 achieved diagnostic accuracy rates of 69.6%, 57.4%, and 64.9%, respectively. However, none of the models exhibited comparable performance in patients under 12 years, with accuracy rates of 20.8%, 20.8%, and 12.5%, respectively. For pneumothorax cases in patients aged over 12, ChatGPT-4o showed moderate response consistency (Kappa = 0.39; 95% CI: 0.28–0.50; p < 0.001), Gemini 2.0 displayed perfect agreement (Kappa = 1.00; 95% CI: 0.89–1.11; p < 0.001), and Claude 3.5 yielded moderate agreement (Kappa = 0.55 and 0.49; p < 0.001).

The application of AI models in medical imaging is becoming increasingly widespread [21]. Clusmann et al. highlighted in their study the potential utility of LLMs in healthcare, particularly for interpreting and reporting radiological images [13]. Similarly, Bhayana et al. assessed ChatGPT’s performance in board-style radiology examinations and provided key insights into the model’s strengths and limitations [22]. In a pilot study by Zhu et al., ChatGPT-4V was evaluated for its ability to interpret radiological images, achieving a diagnostic accuracy of 77% on USMLE-style questions [10]. The same study also showed a significant drop in accuracy—to 19.54%—when detailed clinical information was withheld. Noda et al. reported that ChatGPT’s performance in orthopedic imaging was comparable to that of expert physicians but emphasized its vulnerability to diagnostic errors in the absence of clinical context [14]. Yuan et al. explored the integration of multimodal AI with ultra-high-field MRI and reported a 78.9% accuracy in modality recognition by ChatGPT-4V at 7T MRI [23]. Furthermore, in a study by Liang et al., the Claude 3.5 Sonnet model demonstrated 79.43% accuracy in interpreting imaging modalities related to laryngeal cancer surgery [24]. Noda et al. reiterated that ChatGPT’s performance in orthopedic imaging is comparable to experts, while also being susceptible to errors when clinical context is lacking [25]. Echoing these findings, our study also found that ChatGPT-4o, Gemini 2.0, and Claude 3.5 demonstrated radiological interpretation accuracies of 69.6%, 57.4%, and 64.9%, respectively.

When the diagnostic performance of the models was analyzed by age group, significant differences were identified. In patients over 12 years of age, ChatGPT-4o showed significantly higher strict accuracy than Gemini 2.0 (p = 0.021), whereas in patients under 12, Claude 3.5 outperformed ChatGPT-4o in terms of overall accuracy (p = 0.008). This discrepancy may be attributable to anatomical and radiographic differences between pediatric and adult pneumothorax imaging, as well as additional factors. Lower accuracy in pediatric cases, in particular, may stem from the following considerations. First, flexible rib cages and smaller lung volumes in children can make the pleural separation less distinct on chest X-rays, making detection more challenging. Second, pediatric X-rays are often obtained with lower radiation doses for safety, resulting in lower image contrast. Finally, because pediatric pneumothorax is relatively rare, such cases are underrepresented in model training datasets, limiting the model’s ability to recognize them reliably. In a study by Noda et al., ChatGPT exhibited variation in diagnostic performance across age groups in the context of orthopedic image interpretation [25]. Similarly, Yuan et al. emphasized that the diagnostic performance of LLMs can vary across patient populations, particularly due to limited training data tailored to specific cohorts [23]. Zhu et al. further proposed that with sufficient training data, multimodal LLMs could achieve higher diagnostic accuracy across diverse patient populations [10]. The lower accuracy observed in the under-12 group may stem from the relative rarity of pediatric pneumothorax cases and their underrepresentation in BDM training datasets.Another notable finding of our study was the relationship between pneumothorax size and diagnostic accuracy. Among patients over 12 years, ChatGPT-4o demonstrated significantly higher strict accuracy in the large pneumothorax group compared to the small group (81.6% vs. 42.2%; p < 0.001). Likewise, Gemini 2.0 achieved significantly higher ideal accuracy in the large pneumothorax group (p = 0.019). These findings suggest that LLMs are more effective at detecting radiographically prominent abnormalities. As shown in the study by Güneş and Ülkir, visual diagnostic performance of LLMs improves in proportion to the conspicuity of the pathology under examination [14]. Similarly, Zhu et al. reported that subtle lesions in radiographic images are more challenging for LLMs to identify, whereas prominent findings are recognized with higher accuracy [10]. This may reflect the greater prevalence of clear pathologies in training datasets. Additionally, since these technologies are still evolving, their ability to detect subtle abnormalities may currently be limited.

Another important finding concerns the internal consistency of the models. ChatGPT-4o exhibited moderate consistency in the large pneumothorax group but low consistency in the small group (Kappa = 0.39 vs. 0.04). Gemini 2.0, by contrast, demonstrated perfect consistency in the large pneumothorax group (Kappa = 1.00). Huppertz et al. also reported that GPT-4 V’s consistency varied over time, with performance degrading in the long term [26]. Yuan et al. observed that LLMs can exhibit fluctuating response consistency, particularly in complex cases [23]. This variability may stem from the stochastic nature of LLMs and the probabilistic weighting of parameters during each generation. Zhang et al. emphasized that consistency is a critical prerequisite for the clinical reliability of AI-driven imaging systems [9]. Given that consistency is essential in clinical decision-making, further improvements in this area are necessary before LLMs can be reliably integrated into clinical practice.

The potential clinical applications of LLMs in the field of medical imaging offer significant opportunities, particularly in emergency settings where rapid diagnosis is critical. Güneş and Ülkir emphasized the importance of timely recognition of life-threatening conditions such as pneumothorax through rapid radiologic interpretation in emergency departments [14]. In our study, the notably high diagnostic accuracy of ChatGPT-4o for large pneumothorax cases (81.6%) suggests its potential utility as a preliminary screening tool in emergency care. Similarly, Adriany et al. reported that AI-assisted analysis systems used in high-field imaging can accelerate the diagnostic process and enhance accuracy when integrated into clinical decision support frameworks [27]. In this context, the performance of LLMs in our study suggests that their application in emergency departments could help optimize diagnostic and therapeutic workflows. In clinical practice, the potential utility of LLMs may lie in their role as adjunctive tools for preliminary screening, particularly in emergency settings where rapid detection of large pneumothoraces is critical. Their integration into decision-support workflows, with radiologist confirmation for subtle or equivocal findings, could optimize patient triage and expedite management. Future investigations should also address the cost-effectiveness of integrating LLMs into clinical workflows, as economic feasibility will be a key determinant of widespread adoption.Our findings demonstrate that the latest LLMs—ChatGPT-4o, Gemini 2.0, and Claude 3.5—have achieved meaningful progress in evaluating radiologic images in cases with basic clinical complaints. One strength of this study is the comparative evaluation of these models across various age groups and pneumothorax sizes. However, several limitations should be acknowledged. First, it remains unclear to what extent pneumothorax images were represented in the training datasets of the LLMs utilized. Second, the resolution and quality of the radiographic images used in this study may have influenced the models’ performance. Third, the study did not include a control group of chest radiographs without pneumothorax, which limits the ability to assess the specificity of the models and their ability to distinguish pneumothorax from other pathologies. Fourth, as our dataset exclusively comprised CT-confirmed pneumothorax cases, classical false positive rates could not be determined. Some instances involved underestimation or misclassification of pneumothorax size, particularly in subtle or small cases; however, these do not equate to conventional false negatives. Future prospective studies incorporating chest radiographs without pneumothorax as a control group will be necessary to allow accurate estimation of both false positive and false negative rates. Lastly, the LLMs evaluated represent a rapidly evolving technological domain; their continuous updates may result in variability in performance over time.

In addition to technical limitations, legal and ethical considerations must also be acknowledged. The use of LLMs in clinical practice raises important questions regarding patient data confidentiality, responsibility for potential diagnostic errors, and malpractice liability. Therefore, these models should only be applied as adjunctive tools within a regulated framework, with ultimate responsibility remaining with licensed physicians.Beyond clinical applications, LLMs may also hold promise as educational tools. By providing simulated case scenarios and radiologic interpretation exercises, they could support medical students and junior clinicians in developing diagnostic reasoning skills. Nevertheless, their use in education should complement, not replace, expert-led training.

## 5. Conclusion

In conclusion, this study evaluated the diagnostic performance of three different LLMs in identifying pneumothorax and demonstrated that ChatGPT-4o achieved the highest overall accuracy. Age group and pneumothorax size were identified as key factors influencing model performance. These findings highlight the potential utility of LLMs in supporting radiologic diagnosis in emergency care settings. Furthermore, integrating such models into clinical decision-making and using them as assistive tools for radiologists and clinicians may contribute to the advancement of AI applications in healthcare. However, before widespread clinical implementation, these models should be prospectively validated in broader and more diverse patient populations, and standardized validation protocols must be developed.

## Supporting information

S1 File Dataset. (XLSX)

S2 File SPSS data file for statistical analyses. (SAV)
